# When helping is risky: The influence of ethical attributes on consumers’ willingness to buy farmer-assisting agricultural products online

**DOI:** 10.3389/fpsyg.2022.1014972

**Published:** 2022-11-02

**Authors:** Jingjing Wu, Chao Wang, Yingzheng Yan, Qiujin Zheng

**Affiliations:** ^1^College of Management and Economics, Fujian Agriculture and Forestry University, Fuzhou, China; ^2^College of Forestry, Fujian Agriculture and Forestry University, Fuzhou, China; ^3^School of Journalism and Communication, Minjiang University, Fuzhou, China

**Keywords:** ethical attributes, collective efficacy, risk perception, farmer-assisting agricultural products, online shopping

## Abstract

Chinese e-commerce platforms have long helped to sell agricultural products through farmer-assisting marketing activities, effectively alleviating the problem of stagnant agricultural products in some areas, and have become a valuable cause-related marketing strategy. The ethical attributes of farmer-assisting agricultural products have unique value compared with other agricultural products. However, the existing research rarely pays attention to the influence of the ethical attributes of farmer-assisting agricultural products on consumers’ willingness to buy farmer-assisting agricultural products online. Based on collective efficacy theory and risk perception theory, this study systematically explores the influence mechanism of ethical attributes (symbolic ethical attribute and functional ethical attribute) on consumers’ willingness to buy farmer-assisting agricultural products online through three situational experiments. The results show that compared with functional ethical attributes, symbolic ethical attributes have a more positive impact on consumers’ willingness to buy farmer-assisting agricultural products online. In addition, it further reveals two mediating pathways of ethical effects (collective efficacy and risk perception) and boundaries (emergency of farmer-assisting events). This study helps to understand the ethical attributes of farmer-assisting agricultural products, and provides some practical suggestions for e-commerce enterprises implementing farmer-assisting marketing communication activities or marketers developing and promoting farmer-assisting agricultural products.

## Introduction

Unlike traditional promotions, e-commerce-assisted agricultural marketing is an innovative model that links the social support of e-commerce platforms to the sales of farmer-assisting agricultural products (Lee and Charles, 2021). In fact, since the outbreak of the COVID-19 pandemic, Chinese e-commerce platforms have helped sell agricultural products through farmer-assisting marketing activities, effectively alleviating the problem of stagnant agricultural products in some areas and reducing the impact of the epidemic on farmers’ income loss (Gu and Wang, 2020). In 2021, the e-tailing volume of agricultural products recorded in China reached 422.1 billion yuan, up 2.8% year on year, but only accounted for 4% of the e-tailing volume of physical goods, said a report on E-Commerce in China (2021) released by the Chinese Ministry of Commerce. The above data fully demonstrates that although the e-commerce of agricultural products in China is gradually developing, its penetration rate is still relatively low. Thus, if we want to promote growth in rural incomes further, it is essential to fully utilize the active role of digital technology in rural revitalization. In face of the COVID-19 pandemic, e-commerce-assisted agricultural marketing has become a valuable cause-related marketing strategy, which can not only sell stagnant agricultural products but also force rural advantageous and characteristic industries to become bigger, stronger, and better, and develop into an important channel for online sales of agricultural products (Xiao et al., 2021). In terms of e-commerce-assisted agricultural marketing, this study also refers to the relevant research on cause-related marketing (Guerreiro et al., 2016).

E-commerce-assisted agricultural marketing has received more and more attention and support (Li et al., 2022). It is essentially a moral economic behavior that people’s purchasing behavior is imbued with good moral value by encouraging consumers to buy agricultural products, with the help of moral mobilization (Mai and Li, 2014; Han et al., 2018). Thus, the purchase of farmer-assisting agricultural products by consumers is essentially a kind of “ethical consumption” (Quan, 2021). Ethical consumption is defined as a type of product purchased by consumers that contains not only direct use value but also other attribute values (such as social development or environmental sustainability; Long and Murray, 2013). Wang et al. (2021) revealed that ethical attributes are the main motivation for consumers to buy agricultural products online from poor areas and to pay more. Thus, ethical attributes are a potential influence on consumers’ purchase of farmer-assisting agricultural products, and this study uses the term “ethical effect” to refer to this influence. This effect is similar to earlier studies on prosocial behavior, green consumption, and fair-trade consumption, but these studies also point to the complexity of consumers’ consumption decisions for products with ethical attributes (Herédia-Colaço and Coelho do Vale, 2018). The studies by Bodur et al. (2014) and Herédia-Colaço and Coelho do Vale (2018) have emphasized the importance of alignment of ethical attributes and product category interests. Customers could be worried about buying things with moral qualities that fall short in terms of utility and quality for normal use (Mai et al., 2019). However, unlike the general type of ethical commodities previously investigated, farmer-assisting agricultural products not only satisfy the private utility value of consumer health and safety but also reflect the public utility value of assisting struggling farmers (Wang et al., 2021; Zeng Q. Y. et al., 2021). As a result, earlier research on ethical consumption motives for general types of products is no longer useful in assessing and forecasting consumer purchase decision problems involving farmer-assistance agricultural products. Agriculture products are the most important component in the e-commerce-assisted agriculture marketing scenario for gaining market recognition, and it is necessary to explore further the deeply embedded psychological mechanisms and theoretical logic of the influence of their ethical attributes on consumers’ decisions to purchase assisted agriculture products online.

In the past, most scholars have focused on the psychological variables of the individual level of philanthropists to research ethical consumption issues. For example, philanthropists formed positive ethical consumption attitudes, willingness, or behaviors based on psychological factors such as ethical values, empathy, guilt relief, or personal cost-effectiveness (Yang et al., 2014; Savary and Goldsmith, 2020). However, Fritsche et al. (2018) questioned the individualistic view and emphasized that the collective dimension of ethical consumption should be addressed. When consumers are concerned about collective goals, demand for social welfare products increases (Simpson et al., 2021), especially in the network, the positive dynamic events can enhance the network collective behavior intention through the digital emotion infection (Goldenberg and Gross, 2020; Xie and Li, 2022). Thus, although some studies have indicated that the e-commerce platform has the potential for farmer-assisting and poverty alleviation (Peng et al., 2021; Li et al., 2022; Zeng et al., 2022), few scholars consider the role of social media communication in the psychological process of consumers’ online shopping for farmer-assisting agricultural products. In particular, there are few related studies on collective efficacy based on e-commerce platform (Hornsey et al., 2021; Lee and Littles, 2021; Valizadeh et al., 2022). This study argues that the concept of collective efficacy is an essential supplement to ethical consumption research. It is necessary to further study the potential mechanism of the influence of ethical attributes on consumers’ willingness to buy farmer-assisting agricultural products online from the perspective of collective efficacy.

Furthermore, in online retail, the physical distance between the online retailer and the consumer generates Internet ethics concerns among consumers (Lee and Charles, 2021). As an illustration, some online retailers “trap” consumers through false and exaggerated “tragic marketing” tricks (Zhou, 2020). Fresh agricultural products are relatively non-standardized products, and quality issues such as shoddy products and inconsistent pictures exacerbate risk concerns in the e-commerce environment (Fan and Liu, 2021). Previous research has found that the degree to which consumers are willing to take risks also affects their willingness to help others when doing so may expose them to certain risks (Gangadharan et al., 2019; Beaud et al., 2022; Costa et al., 2022). Consumers’ decision to buy farmer-assisting agricultural products with ethical attributes online is complicated by the potential conflict of interest between individual goals (immediate product benefits) and long-term collective goals (contributing to social well-being).

The significance of a product’s ethical attributes for marketers seeking to differentiate their products cannot be overstated, as acknowledged by the vast majority of academics (Iweala et al., 2019; Hindsley et al., 2020; Quan, 2021). However, it is crucial to consider what ethical attributes agricultural products should have in order to increase consumers’ willingness to buy farmer-assisting agricultural products online. What is the underlying mechanism behind the ethical effect? Where are the boundaries of ethical effect? To address the above issues, this study focuses on the special nature of farmer-assisting agricultural products themselves (i.e., ethical attributes), and explores the differences between different ethical attributes (symbolic and functional) on the intrinsic drivers in the formation of consumers’ online purchase decisions through the introduction the collective efficacy theory and risk perception theory. Simultaneously, various scenarios of the urgency of farmer-assisting events are introduced in order to identify clear boundaries.

The study is innovative in three aspects. First, in cause-related marketing activities, scholars have explored the impact on consumer behavioral decisions from marketing strategies such as victim’s image (Homer, 2021; Zhou et al., 2021), victim’s recognizability (Hou et al., 2022), and advertising personalization (Kim and Kim, 2022). However, few studies have focused on product attributes. This study broadens the research viewpoint on cause-related marketing by examining the relationship between the ethical attributes of farmer-assistance agricultural products and consumers’ willingness to buy online. This study distinguishes between symbolic and functional ethical attributes and compares them. Incorporating collective efficacy theory and risk perception theory clarifies the psychological mechanisms by which the two ethical attributes effect consumers’ willingness to purchase agricultural products online. Second, this study identified the importance of collective efficacy and risk perception for consumers to understand the significance of e-commerce-assisted agricultural marketing activities, thereby contributing to relevant research on collective efficacy and risk perception theories. Third, the moderating effect of the urgency of farmer-assisting events (sudden disaster scenario and normative difficulty scenario) on the relationship between ethical attributes and consumers’ online purchase decisions was investigated. The interaction of the two has a significant effect on customers’ willingness to buy farmer-assisting agricultural online, so complementing prior research on consumers’ ethical consumption. In summary, the findings of this study provide a necessary theoretical basis for e-commerce platforms to effectively develop farmer-assisting marketing strategies, thus helping agricultural products access the market and enhancing farmers’ income, as well as rural revitalization.

## Theoretical backgrounds

### Ethical attributes of farmer-assisting agricultural products

Ethical attributes refer to product attributes that reflect social fairness, environmental protection, and other ethical issues. Bezençon et al. (2020) have revealed that purchasing products with ethical attributes (e.g., EcoConscious or No Child Labor statement) can reflect their concerns and support for social or ethical issues, but has nothing to do with the function of the product itself. Thus, ethical attributes also refer to symbolic ethical attributes to a certain extent (Peloza et al., 2013; Sama et al., 2018; Hindsley et al., 2020; Tofighi et al., 2020; Das et al., 2021; Wang et al., 2021; Salam et al., 2022; see Table 1). Unlike previous research, Bodur et al. (2014) believed that products with ethical attributes also have functional benefits at the same time. Ethical attributes can generally be divided into functional ethical attributes (such as product performance, safety, etc.) and symbolic ethical attributes (such as public welfare, social responsibility, etc.; Bodur et al., 2014; Tofighi et al., 2020).

**Table 1:** Literature on ethical attributes.

Reference	Topic	Ethical attributes	Ethical-related intention
Peloza et al. (2013)	Green marketing	Environmental sustainability	Product preference
Sama et al. (2018)	Fair trade	Socio-environmental responsibility	Willingness to pay premiums
Hindsley et al. (2020)	Direct-trade coffee	Farmers receive a premium amount，harvesting method is sustainable	Willingness to pay premiums
Tofighi et al. (2020)	Sustainable brands	Positive implications for the environment, human rights, social issues, and animal welfare	Brand evaluations
Das et al. (2021)	Gift-giving	Made of eco-friendly ingredients	Purchase intention
Wang et al. (2021)	Poverty alleviation consumption	Apples from poverty-stricken areas	Willingness to pay
Salam et al. (2022)	Organic food consumption	Ethical production	Purchase intention
Previous studies	Focus on standardized products consumption	Focuses on symbolic benefits of ethical attributes	
This paper	Non-standardized fresh produce consumption	Focus on symbolic ethical attributes and functional ethical attributes	Consumers’ willingness to buy farmer-assisting agricultural products online

Consumers’ purchasing decisions are based on evaluations and needs for product-specific attributes. A necessary condition for promoting e-commerce-assisted agricultural marketing in consumer groups is that consumers have an effective demand for farmer-assisting agricultural products. This demand depends on whether farmer-assisting agricultural products can bring special value to consumers compared with traditional e-commerce agricultural products. Thus, according to the difference in the characteristics of ethical attributes, consumers’ demand for farmer-assisting agricultural products can be divided into two situations: on the one hand, farmer-assisting agricultural products convey the message of “functional ethical attributes,” emphasizing the advantages of naturalness, safety and taste, and meeting consumers’ practical needs in terms of functionality. The common perception is that the land in remote mountainous areas is fertile, the environment is not polluted, the production conditions are more traditional, and the products are healthier and more nutritious (Wang et al., 2018). On the other hand, farmer-assisting agricultural products convey the message of “symbolic ethical attributes,” emphasizing that the products have the characteristics of helping trapped or impoverished farmers, and can meet the emotional needs of consumers in terms of morality and emotion. Zeng Q. Y. et al. (2021) revealed that helping farmers is the most common motivation for consumers to buy farmer-assisting agricultural products.

In general, most of the existing research on the ethical attributes of farmer-assisting agricultural products is still limited to the symbolic value of the product, ignoring the functional value of the product such as safety and taste. Thus, further systematic analysis of the ethical attributes of farmer-assisting agricultural products is required.

### Collective efficacy

Collective efficacy originates from psychology and sociology and is based on the development of self-efficacy theory. Bandura (2002) defined collective efficacy as an individual’s belief in the collective ability of the collective to effectively achieve a goal. Thus, collective efficacy is not the ability of the group itself, but the individual’s perception of the collective ability. When a majority of the group expresses support and appreciation for prosocial activities, individuals respond to others’ prosocial behavioral expectations by adjusting their behavioral willingness to participate in environmental protection activities (Bamberg et al., 2015), plastic reduction (Reese and Junge, 2017), and recycling activities (Wang et al., 2020), etc.

Collective efficacy is considered one of the critical factors in collective activity research, and its importance has been explored in research contexts such as community activity (Carbone and McMillin, 2019), environmental protection (Higham et al., 2019; Hornsey et al., 2021), organizational leadership (Valizadeh et al., 2022), and political activity (Chapman et al., 2022). Valizadeh et al. (2022) pointed out that collective efficacy has a significant positive impact on social resilience in the COVID-19 pandemic. Zhao et al. (2022) also indicated that during the epidemic, people’s participation efficacy motivates them to participate in online collective activities. Although there are few studies on collective efficacy in the context of e-commerce-assisted agricultural marketing, the above studies confirm the role of individuals’ expected outcomes of collective power in stimulating pro-environmental actions and social participation intentions. Further, the study by Simpson et al. (2021) states that when the collective efficacy of crowdfunding participants is high, they will be motivated to pursue and accomplish goal outcome that benefits the collective. The results of this study also indicated that collective efficacy enhances consumer demand for socially beneficial products but does not affect the demand for self-interested products. Similarly, Li and Sun (2022) showed that altruistic appeals in green product advertising can motivate consumers to increase their purchase intentions when they are concerned about collective interests. Thus, collective efficacy theory provides a valuable perspective on how people view the ability and effectiveness of actions to solve the problem of farmer-assisting and poverty alleviation. Specifically, e-commerce-assisted agricultural marketing can enhance the demand for farmer-assisting agricultural products by increasing consumers’ attention to collective goals. Based on the definition of Doran and Hanss (2022), this study defines the collective efficacy of farmer-assisting consumption as people’s perception of the collective’s ability to successfully help farmers solve the problem of stagnant agricultural products.

### The urgency of farmer-assisting events

In cause-related marketing activities, consumers’ judgment of the urgency of causal-related events will affect their attitudes and behavioral decisions (Luo and Lv, 2019). Zheng et al. (2019) divided cause-related events into two scenarios according to their urgency: long-term difficulty (e.g., poverty, pollution) and sudden disaster (e.g., epidemic, earthquake). People are more likely to donate to sudden disasters than to long-term difficulties. According to the attribution theory, when a company provides help to the people suffered a sudden disaster, not only the uncontrollable and unpredictable nature of the disaster can prevent people from blaming the victim, but also the company’s behavior will also increase the consumer’s sense of identity (Vanhamme et al., 2012; Chang et al., 2021). When a company conducts cause-related marketing campaigns on long-standing problems, it often leads consumers to associate with the company’s self-interested purpose (such as improving performance or reputation; Kuo and Rice, 2015; Zhang et al., 2020; Chang et al., 2021). Thus, the urgency of farmer-assisting events may affect consumers’ decision-making on ethical behavior to a certain extent. Combined with previous studies on the urgency of cause-related events, this study also divides the urgency of farmer-assisting events into sudden disasters and normative difficulties. A sudden disaster scenario refers to carrying out farmer-assisting activities for the problem of stagnant agricultural products caused by sudden disasters; a normative difficulty scenario refers to carrying out farmer-assisting activities for the long-term disharmony between the production and marketing of agricultural products in poor areas.

## Research hypotheses

### Ethical attributes and consumers’ willingness to buy farmer-assisting agricultural products online

Ethical attributes play a crucial role in consumers’ ethical consumption (Bezençon et al., 2020; Tofighi et al., 2020; Das et al., 2021). Both symbolic ethical attributes and functional ethical attributes can bring utility to consumers (Zeng Q. Y. et al., 2021). Herédia-Colaço and Coelho do Vale (2018) and Iweala et al. (2019) found that organic products with symbolic ethical attributes (e.g., environmental protection, animal welfare) lead to higher ratings by consumers. Thus, the symbolic ethical attributes of products are considered to be an important factor affecting the ethical consumption of consumers. With the improvement of consumers’ awareness of food safety, the actual demand for high-quality agricultural products is also increasing. The natural health attributes and local characteristics of farmer-assisting agricultural products perfectly fit the consumer’s interest motives (Zeng Y. C. et al., 2021). In particular, self-interest motivation based on natural health attributes has repeatedly appeared in consumer purchasing decision research (Chekima et al., 2017; Zhou et al., 2017). On that basis, this study believes that the two types of ethical attributes will have a positive impact on consumers’ willingness to buy farmer-assisting agricultural products online.

Further, some scholars have indicated that consumers also face attribute trade-offs when purchasing ethical products with multiple attributes (Araque-Padilla et al., 2015). When products are rated low, symbolic ethical attributes are more likely than functional ethical attributes (or other attributes) to give consumers the pleasure and satisfaction derived from the perceived assessment of contributing to the well-being of society (Bezençon et al., 2020). For example, in the fresh products e-commerce scenario, the uncertainty of the quality and standardization of fresh agricultural products also further leads to lower overall consumer evaluation of online agricultural products. Thus, compared with functional ethical attributes, the symbolic ethical attributes of farmer-assisting agricultural products can more directly convey the social benefits of farmer-assisting to consumers, thereby increasing consumer evaluation and willingness to purchase online. Based on the above analysis, this study proposes the following hypothesis:

*H1*: Ethical attributes influence consumers' willingness to buy farmer-assisting agricultural products online, and symbolic ethical attributes have a more positive impact on consumers' willingness to buy farmer-assisting agricultural products online compared to functional ethical attributes.

### Ethical attributes and consumers’ willingness to buy farmer-assisting agricultural products online: The mediating effect of collective efficacy

Consumers’ participation in cause-related marketing activities is not only a prosocial behavior but can also be considered a collective activity (Hou et al., 2022). The study by Alavi and McCormick (2018) showed that collective efficacy determines an individual’s attempts and efforts to engage in collective activities. For example, Doran and Hanss (2022) found that sustainable environmental protection actions can only be promoted when people recognize the value of collective efforts. Thus, as a type of collective activity, farmer-assisting consumption requires not only individual efforts but also collective efforts to jointly address.

Based on social identity theory, Arslanagic-Kalajdzic et al. (2022) discovered that moral appeals related to social issues will activate cohesion within consumer groups and increase the likelihood of positive behavioral responses, such as generating purchase intentions. van Zomeren et al. (2012) combined moral beliefs with a social identity model of collective activity, and pointed out that moral beliefs positively affect collective efficacy and can predict collective activity intentions. Similarly, this study infers that if farmer-assisting agricultural products convey ethical appeal and ethical value information to consumers, it can activate the sense of identity of individuals and groups, thereby forming collective efficacy. Although both types of ethical attributes of farmer-assisting agricultural products demonstrate the benefits of participating in activities, symbolic ethical attributes are designed to stimulate the moral feeling of helping others, while functional ethical attributes emphasize the practical utility of self-interest. Thus, symbolic ethical attributes are more in line with the public’s ethical values, attract online audiences more widely, and form collective efficacy. Based on the above analysis, the following hypotheses are made:

*H2a*: Ethical attributes affect consumers' collective efficacy, and symbolic ethical attributes can stimulate consumers' collective efficacy more than functional ethical attributes.

*H2b*: Collective efficacy plays a mediating role between ethical attributes and consumers' willingness to buy farmer-assisting agricultural products online.

### Ethical attributes and consumers’ willingness to buy farmer-assisting agricultural products online: The mediating effect of risk perception

E-commerce-assisted agricultural marketing is a new scenario extended by the combination of cause-related activities and fresh online shopping. Compared with traditional offline cause-related marketing, consumers have information asymmetry before receiving goods, mainly facing risks in information search, product quality, logistics, and transportation, as well as after-sales service (Zhang et al., 2015), which also hinders consumers from frequently purchasing fresh agricultural products online to some extent (Zheng et al., 2020; Wu et al., 2022).

Scholars have pointed out that ethical attributes may come with liability, especially in terms of product quality, to mitigate consumer risk perceptions to some extent (Bodur et al., 2014). Xue et al. (2022) pointed out that the green image of the platform reflects the platform’s commitment to social responsibility and sustainable development, which is conducive to enhancing consumer identification and reducing concerns. Thus, symbolic ethical attributes can, to a certain extent, improve product credibility and mitigate consumer risk perceptions. However, some scholars also found that it is not always beneficial for products to have ethical attributes. Based on zero-sum heuristic research, consumers may equate a product’s ethical attributes with its lower functional attributes (Chernev, 2007; Mai et al., 2019), i.e., positioning an ethical product as a “humanitarian” but less functional product. A typical example is when a functional product (such as laundry detergent) is labeled as ethical (compared to no label), consumers will use a larger amount (Lin and Chang, 2012). Quan (2021) emphasized that although farmer-assisting agricultural products convey information about functional ethical attributes such as health and safety to consumers, concerns about product quality may affect purchase intentions to some extent due to the low level of consumers’ overall knowledge about the quality and function of cause-related products. Thus, this study argues that consumers’ attitudes toward the farmer-assisting agricultural products with functional ethical attributes tend to be based on more rational or practical considerations, resulting in more risk perceptions. In contrast, the symbolic ethical attribute of farmer-assisting agricultural products is conducive to stimulating positive associations among consumers, thus reducing risk perceptions. Based on the above analysis, the following hypotheses are proposed:

*H3a*: Ethical attributes affect consumers' risk perception, and symbolic ethical attributes can alleviate consumers' risk perception more than functional ethical attributes.

*H3b*: Risk perception plays a mediating role between ethical attributes and consumers' willingness to buy farmer-assisting agricultural products online.

### The moderating effect of the urgency of farmer-assisting events

External environmental issues have a significant moderating effect on the relationship between consumers’ cognitive values and ethical consumption willingness (Kushwah et al., 2019). Thus, consumers’ cognition and value judgment on the characteristics of ethical attributes will be greatly affected by the urgency of the farmer-assisting event. Specifically, the study by Hou et al. (2022) showed that consumers tend to provide more help for tangibly identifiable individuals or events than for unidentified or normative events. That is, consumers are more responsive to a single sudden disaster scenario. Further, sudden disasters receive more social media coverage and appeals than ongoing difficulties, which tend to resonate with the public and create a resonance effect (Vanhamme et al., 2012). For example, the epidemic has rapidly driven the social ethics of “one side in trouble, all sides support,” and the general public voluntarily joins and establishes emergency organic relationships under the social media “positive energy” propaganda (Zhang and Meng, 2021). At the same time, the real-time dynamic information delivered by social media further helps consumers understand the progress and effectiveness of the emergency, activating some positive cognitive evaluation while alleviating risk concerns (Mirbabaie et al., 2021). However, in an emergency, if the information involves functional factors, it will increase the sensitivity and vigilance of consumers (Liu et al., 2022). Based on this, this study argues that in sudden disaster scenarios, symbolic ethical attributes are more effective in enhancing consumers’ ethical emotions, and communication and interaction through social media can further enhance collective efficacy and mitigate risk perceptions, thus promoting consumers’ willingness to buy farmer-assisting agricultural products. However, as the scale of the group in need of help increases, consumers will perceive an increase in the difficulty of helping (Sharma and Morwitz, 2016). In contrast, in a normative difficulty scenario, farmer-assisting events will not attract strong social attention and consumers will not be able to see the effect of farmer-assisting in the short term (Salam et al., 2022), thus ignoring the difference between the two types of ethical attributes to some extent. Thus, there is no significant difference between the collective efficacy and risk perceptions inspired by symbolic and functional ethical attributes. Based on the above analysis, this study proposes the following hypotheses:

*H4*: The urgency of farmer-assisting events moderates between ethical attributes and collective efficacy. When in a sudden disaster scenario, symbolic ethical attributes elicit more collective efficacy among consumers than functional ethical attributes. When in a normative difficulty scenario, there is no difference in the collective efficacy induced by functional ethical attributes and symbolic ethical attributes.

*H5*: The urgency of farmer-assisting events moderates between ethical attributes and risk perception. When in a sudden disaster scenario, symbolic ethical attributes mitigate consumers' risk perceptions more than functional ethical attributes. When in a normative difficulty scenario, functional ethical attributes do not differ from symbolic ethical attributes in terms of consumers' risk perceptions.

**Figure 1:**
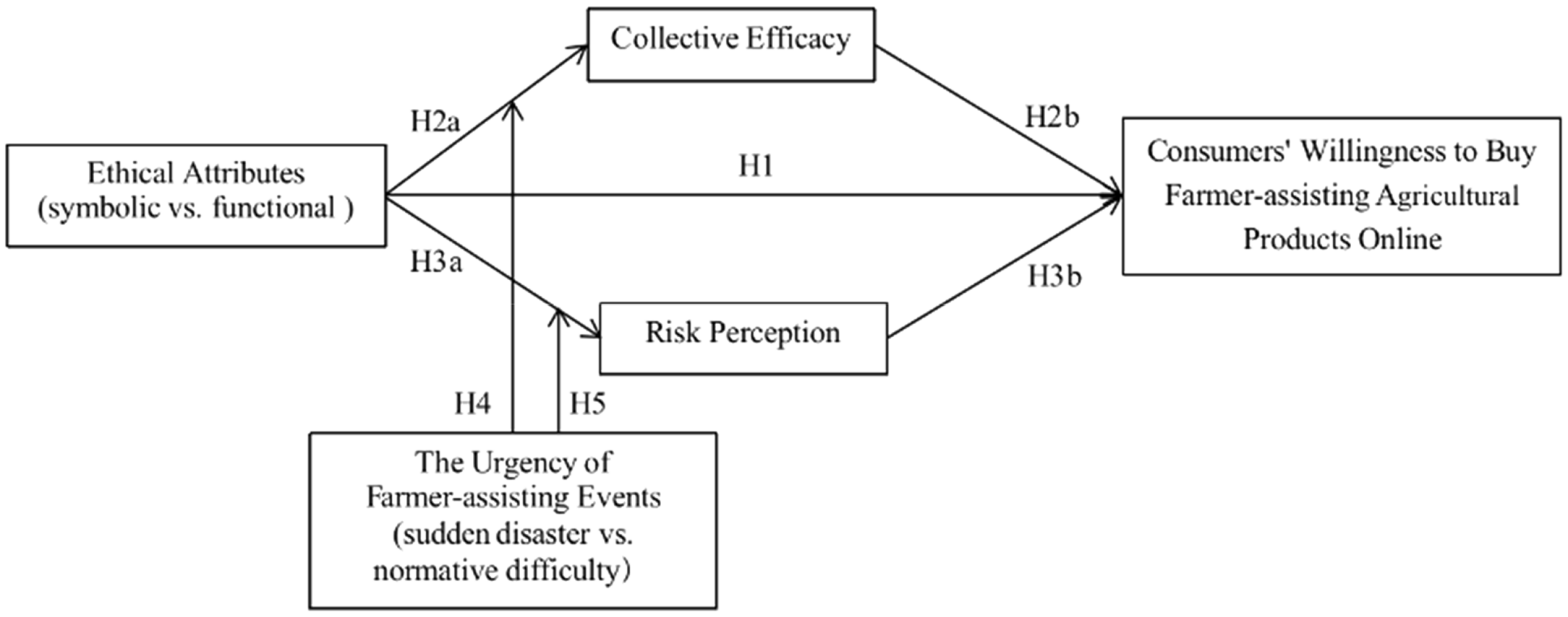
Hypothetical model diagram.

Based on the above theoretical basis and discussion, this study proposes a research model on the influence of ethical attributes on consumers’ willingness to buy farmer-assisting agricultural products online. The details are as follows (see Figure 1).

## Study design and results analysis

This study used an online behavioral experiment research method to verify the causal relationship between the independent and dependent variables. Expressly, we set up an experimental scenario of selling farmer-assisting agricultural products on an e-commerce platform. Subjects were asked to view different information and pictures of farmer-assisting agricultural products and play the role of consumers in making online purchase choices. Finally, we compare the differences in consumers’ willingness to buy farmer-assisting agricultural products online between different groups to test the hypothesis. Overall, three experiments were conducted to test these hypotheses (see Table 2). In Study 1, we tested the main effect of ethical attributes on consumers’ willingness to buy farmer-assisting agricultural products online. Through Study 2, we re-validated the main effect and evaluated the mediating role of collective efficacy and risk perception. In Study 3, the moderating effects of the urgency of farmer-assisting events were investigated. The ethical attributes of farmer-assisting agricultural products were manipulated in two different ways: as orange pictures containing “character” images in Study 1; as orange pictures without “character” images in Study 2 and Study 3. The online experiments were conducted on the Credamo platform, which has been accepted in many refereed journals and has resulted in many excellent papers (Huang and Jaideep, 2020; Gai and Puntoni, 2021).

**Table 2:** Outline of the studies.

Effect/hypothesis	Tested in	Subject source	Independent variable manipulation	Variable measurement
Main effect: H1	Study 1	Credamo platform 150 subjects	3 (functional ethical attribute vs. symbolic ethical attribute vs. non-ethical attribute)	Consumers’ willingness to buy farmer-assisting agricultural products online
Mediating effect: H2	Study 2	Credamo platform 110 subjects	2 (functional ethical attribute vs. symbolic ethical attribute)	Consumers’ willingness to buy farmer-assisting agricultural products online; Collective efficacy; Risk perception
Moderating effect: H3	Study 3	Credamo platform 180 subjects	2 (functional ethical attribute vs. symbolic ethical attribute) * 2 (sudden disaster scenario vs. normative difficulty scenario)	Consumers’ willingness to buy farmer-assisting agricultural products online; Collective efficacy; Risk perception

### Pre-test

The purpose of the pre-test is to identify the farmer-assisting agricultural products for formal experiments. Through interviews with 10 consumers who have experience in purchasing farmer-assisting agricultural products, the types of farmer-assisting agricultural products with high familiarity with the e-commerce platform have been preliminarily determined, including fruits such as oranges and apples, and vegetables such as potatoes and garlic. Furthermore, 30 respondents were randomly selected using a convenience sample questionnaire to answer the question “How familiar are you with the following farmer-assisting fruits or vegetables” (1 = “very unfamiliar”; 7 = “very familiar”). The results showed that consumers have the highest familiarity score with farmer-assisting oranges, M = 5.41. Thus, this study selects oranges as the following experimental materials.

### Study 1: The influence of ethical attributes on consumers’ willingness to buy farmer-assisting agricultural products online

#### Pre-experiment

The purpose of the pre-experiment is to test the validity of the experimental stimuli reflecting two dimensions of ethical attributes (functional ethical attributes and symbolic ethical attributes). Referring to the experimental materials and experimental process design of Bodur et al. (2014) and Bezençon et al. (2020), this study adopts a scenario simulation experimental method and the stimuli are in the form of “pictures + text.” Oranges in A area from the Taobao Baba Farm love channel are selected as the material, which has both “health” and “public welfare” ethical attributes.

The pre-experiment randomly selected 30 subjects (%females = 53.3%, M_age_ = 26.690 years, SD = 3.854) through the sample recommendation service of the Credamo platform. First, all the subjects were presented with the same introduction scenario materials, that is, a kind of stagnant oranges (29.9 yuan, 5 catties, 75–85 mm) in A area is noticed on the e-commerce platform. The subjects then randomly watched one of the two sets of materials presented. The first group read the pictorial material describing the functional ethical properties of the stagnant oranges. The specific content is “the original ecological planting of oranges in A area, sweet and moisturizing with no inflammation, rich in vitamin C, good for beauty and skincare.” The second group read pictures and materials that describe the symbolic ethical attributes of the stagnant oranges. The specific content is “the oranges in A area are hard to find a way to sell, and the mountain farmers are growing them with great effort and urgently need your support.” After the subjects read the above materials, they rated the ethical attribute information of the stagnant oranges on the Semantic Difference Scale (1 = “functional ethical attributes,” 7 = “symbolic ethical attributes”). The independent sample t-test results showed that the mean scores of the first group and the second group were significantly different (M_symbolic_ = 5.933, SD = 0.961 vs. M_functional_ = 2.267, SD = 1.486; t(28) = 8.023, *p* < 0.001), and the stimuli in this group were preliminarily considered to be effective. The effect on perceived ethical attributes was further tested by analysis of covariance (ANCOVA) when controlling for the age and gender of the subjects, and the results were consistent with one-way ANOVA. The specific results were no significant effect of gender on ethical attributes [*F*(1, 26) =1.325, *p* = 0.265] and no significant effect of age on ethical attributes [*F*(1, 26) = 1.821, *p* = 0.189; for brevity, this test was conducted for all the following experiments, but the results of this step of the analysis are not presented].

#### Formal experiments

**Figure 2:**
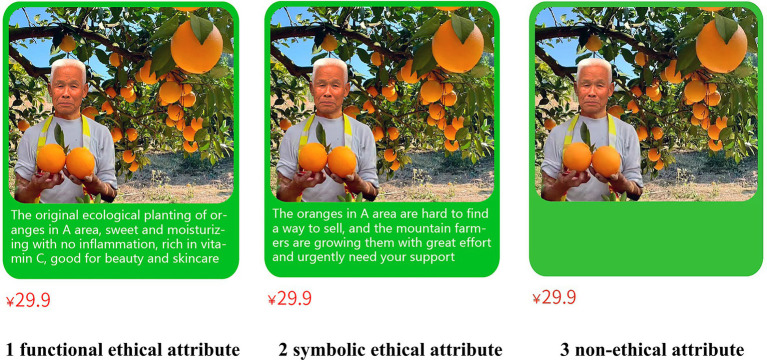
Image of ethical attributes of farmer-assisting agricultural products in Study 1.

The purpose of study 1 is to test the influence of ethical attributes on consumers’ willingness to buy farmer-assisting agricultural products online, i.e., testing hypothesis H1. A single-factor three-level (functional ethical attribute vs. symbolic ethical attribute vs. non-ethical attribute) between-group experiment was designed. The formal experiment recruited 150 subjects (%females = 51.3%, M_age_ = 31.967 years, SD = 6.925) on the Credamo platform, and randomly assigned the subjects to the symbolic ethical attribute group, functional ethical attribute group, and non-ethical attribute group (control group). The three groups of subjects read the experimental materials in the pre-experiment. The experimental materials of the non-ethical attribute group are the same stagnant orange pictures as the ethical attribute group, but there is no ethical attribute text on the picture (see Figure 2). After the subjects read it, they filled in the items of consumers’ willingness to buy farmer-assisting agricultural products online and demographic information. The questionnaire of consumers’ willingness to buy farmer-assisting agricultural products online was adapted from the study of Zhou et al. (2021), with a total of six questions, including “I plan to buy this orange online,” “If necessary, I am willing to buy this orange online,” etc. The question items were assessed using the 7-point Likert scale with 1 = “strongly disagree” and 7 = “strongly agree.” After excluding 30 invalid questionnaires, 40 valid questionnaires were obtained for the functional ethical attribute group, 40 for the symbolic ethical attribute group, and 40 for the non-ethical attribute group (control group).

#### Analysis of results

First, the Cronbach’s alpha value of consumers’ willingness to buy farmer-assisting agricultural products online was 0.788, which was greater than 0.7, and the scale passed the reliability test. Leven’s test showed that *F*(2, 117) = 1.218, *p* = 0.299, further one-way ANOVA can be carried out (All series of experimental analyses of the research passed this test. For the sake of brevity, the following experimental results are omitted to report the data results of this step). Secondly, one-way ANOVA results showed that the influence of symbolic ethical attributes, functional ethical attributes, and non-ethical attributes on consumers’ willingness to buy farmer-assisting agricultural products online was significantly different [*F*(2, 117) = 23.422, *p* < 0.001]. Finally, Turkey’s HSD multiple post-test results showed (see Table 3) that symbolic ethical attribute group (M_difference_ = 0.758, *p* < 0.05) and functional ethical attribute group (M_difference_ = 0.442, *p* < 0.05) were more willing to buy farmer-assisting agricultural products online than non-ethical attribute group (the control group). Furthermore, consumers’ willingness to buy farmer-assisting agricultural products online in symbolic ethical attribute group was significantly higher than that in the functional ethical attribute group (M_difference_ = 0.442, *p* < 0.05). The results of the independent samples t-test also indicate (see Table 4) symbolic ethical attribute group is more likely to increase consumers’ willingness to buy farmer-assisting agricultural products online than the functional ethical attribute group [M_symbolic_ = 6.083, SD = 0.571 vs. M_functional_ = 5.767, SD = 0.392, *t*(78) = 3.792, *p* = 0.002].

**Table 3:** Test results of consumers’ willingness to buy farmer-assisting agricultural products online.

(I) Group	(J) Group	Mean difference (J − I)	SD	95%CI	
				LLCI	ULCI
Non-ethical attribute (control group)	Symbolic ethical attribute	0.758^*^	0.111	−1.023	−0.494
	Functional ethical attribute	0.442^*^	0.111	−0.706	−0.177
Functional ethical attribute	Symbolic ethical attribute	0.316^*^	0.111	0.052	0.581

* *p* < 0.05.

**Table 4:** Results of independent samples *t*-test.

Variable	Factors	Mean value	SD	T	95%CI	
					LLCI	ULCI
Consumers’ willingness to buy farmer-assisting agricultural products online	Symbolic ethical attribute	6.083	0.571	3.133^**^	0.115	0.518
	Functional ethical attribute	5.767	0.392						

** *p* < 0.01.

In conclusion, Study 1 preliminarily proved that compared with the non-ethical attribute, the ethical attribute can significantly improve the willingness of consumers to buy farmer-assisting agricultural products online. Compared with the functional ethical attribute, the symbolic ethical attribute can significantly improve the consumers’ willingness to buy farmer-assisting agricultural products online. Thus, hypothesis H1 is validly verified. In Study 1, the usual marketing stimuli materials, that is, “character” images, were used to meet the real consumption scenarios to a large extent. However, as far as the experimental results are concerned, the presence of “character” images increases the experimental error. Thus, in Study 2, the “character” image in the above stimuli was eliminated, and new stimuli were formed to further explore the mediating effect of collective efficacy and risk perception.

### Study 2: The mediating effect of collective efficacy and risk perception

#### Pre-experiment

The pre-experiment of Study 2 replicated the pre-experiment of Study 1, but the image of farmers was removed from the pictorial material. In this study, 30 subjects (%females = 46.7%, M_Age_ = 25.033 years, SD = 3.843) were randomly selected through the sample recommendation service of the Credamo platform. The results showed that there were significant differences in the material scores of the subjects for symbolic ethical attributes and functional ethical attributes [M_symbolic_ = 5.250, SD = 1.199 vs. M_functional_ = 2.643, SD = 2.061; *t*(28) = 4.108, *p* < 0.001]. Thus, this set of stimuli was proved to be effective.

#### Formal experiments

**Figure 3:**
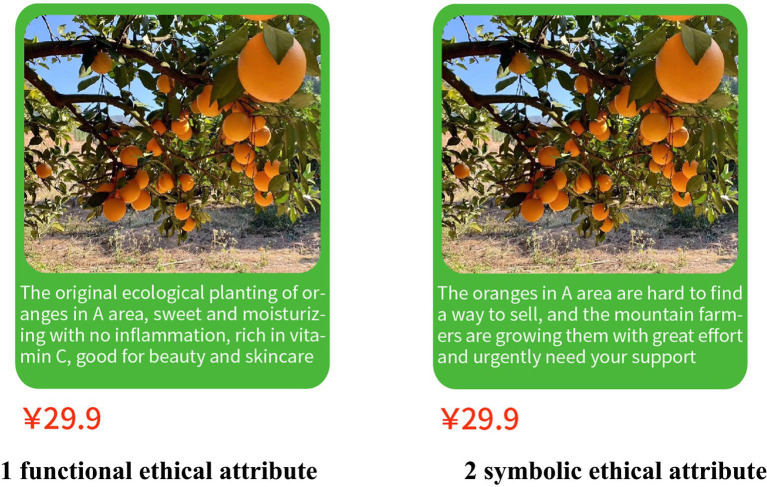
Image of ethical attributes of farmer-assisting agricultural products in Study 2.

The purpose of Study 2 is, first, to revalidate the results of Study 1 and enhance the internal validity of the theoretical model, i.e., to exclude the interference of farmer’s images; second, to test the mediating effects of collective efficacy and risk perception, i.e., to test hypotheses H2a, H2b, H3a, and H3b. A one-way two-level between-group experiment (symbolic ethical attributes vs. functional ethical attributes) was designed. The formal experiment recruited 110 subjects (%females = 50.9%, M_age_ = 28.400 years, SD = 8.190) on the Credamo platform. The subjects were randomly assigned to the symbolic ethical attribute group and the functional ethical attribute group and read the experimental materials in the pre-experiment (see Figure 3). After reading the materials, the subjects were required to fill in the items of consumers’ willingness to buy farmer-assisting agricultural products online, collective efficacy, risk perception, and demographic characteristic. The scale of consumers’ willingness to buy farmer-assisting agricultural products online was the same as in Study 1. The collective efficacy scale was adapted from the study of Doran and Hanss (2022), with four questions, such as “I believe that we as consumers can work together to solve the problem of stagnant oranges” and “I believe that we as consumers can contribute to farmer-assisting and poverty alleviation.” Risk perception was adapted from the study of Zhang et al. (2015), with 4 questions, such as “I am worried that the oranges are inferior and the quality varies,” “I am worried that the oranges will be damaged during transportation,” etc. All scale items were on a 7-point Likert scale, with 1 = “strongly disagree” and 7 = “strongly agree.” After eliminating 30 invalid questionnaires, 40 valid questionnaires were obtained for the functional ethical attribute group and 40 for the symbolic ethical attribute group.

#### Analysis of results

First, a reliability test was performed. The results showed that the Cronbach’s alpha value of consumers’ willingness to buy farmer-assisting agricultural products online was 0.844, the Cronbach’s alpha value of collective efficacy was 0.804, and the Cronbach’s alpha value of risk perception was 0.851, which were all greater than 0.7. Thus, each scale passed the reliability test.

Second, the independent sample t-test was used to verify the main effect of ethical attributes on consumers’ willingness to buy farmer-assisting agricultural products online. The results showed that (see Table 5) compared with functional ethical attributes, consumers showed more willingness to buy farmer-assisting agricultural products with symbolic ethical attributes online [M_symbolic_ = 5.758, SD = 0.406 vs. M_functional_ = 5.358, SD = 0.518, *t*(78) = 3.792, *p* < 0.001], thus the main effect was proved to be significant. The independent sample *t*-test was also used to test the influence of ethical attributes on mediating variables. The results showed that consumers showed higher collective efficacy [M_symbolic_ = 5.404, SD = 0.429 vs. M_functional_ = 5.062, SD = 0.477, *t*(78) = 3.324, *p* < 0.001] and lower risk perception [M_symbolic_ = 4.325, SD = 0.421 vs. M_functional_ = 4.656, SD = 0.392, *t*(78) = 3.596, *p* < 0.001] for the farmer-assisting agricultural products with symbolic ethical attributes. Thus, H1, H2a, and H3a were verified.

**Table 5:** Results of independent samples *t*-test.

Variable	Factors	Mean value	SD	T	95%CI	
					LLCI	ULCI
Consumers’ willingness to buy farmer-assisting agricultural products online	Symbolic ethical attribute	5.758	0.406	3.792^***^	0.190	0.610
	Functional ethical attribute	5.358	0.518			
Collective efficacy	Symbolic ethical attribute	5.404	0.429	3.324^***^	0.137	0.546
	Functional ethical attribute	5.062	0.477			
Risk perception	Symbolic ethical attribute	4.344	0.421	−3.596^***^	−0.515	−0.148
	Functional ethical attribute	4.675	0.392			

*** *p* < 0.001.

**Figure 4:**
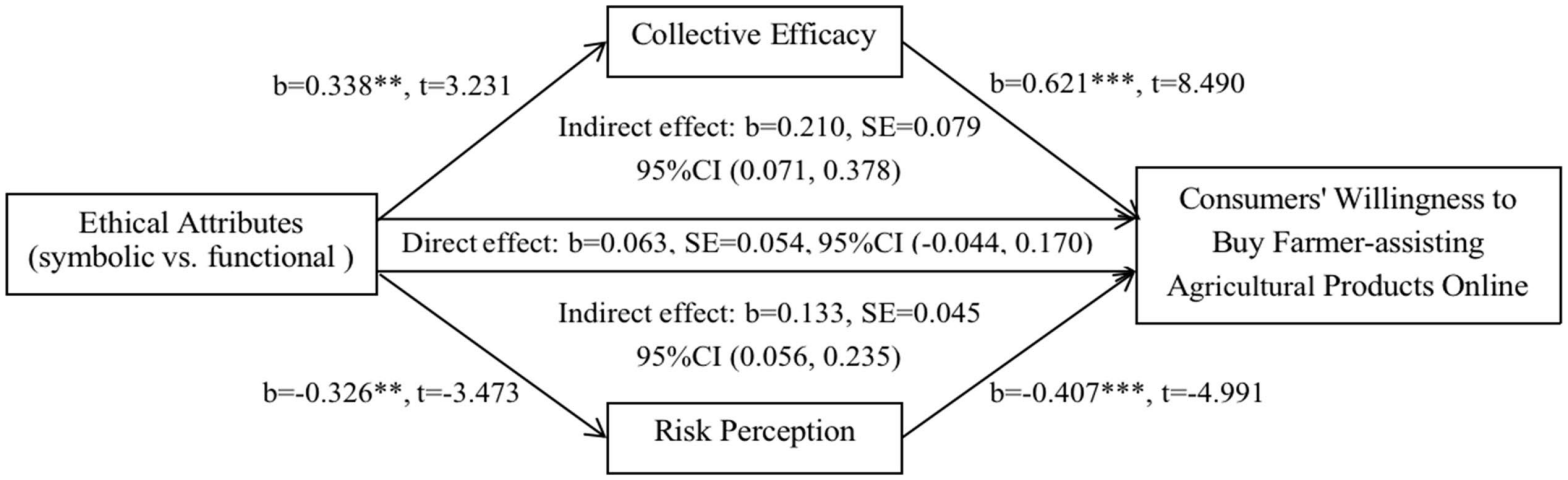
Statistical mediation diagrams for collective efficacy and risk perception. **p < 0.01, ***p < 0.001.

Finally, we examined the mediating effect of collective efficacy and risk perception. Using the process3.0 of the statistical software SPSS22.0, the number of Bootstrap sampling was set to 5,000, the confidence interval was 95%, and model 4 was selected (Hayes, 2013). The results showed that the direct effect of ethical attributes on consumers’ willingness to buy farmer-assisting agricultural products online was not significant (β_direct effect_ = 0.063, 95%CI = [−0.044, 0.170], including 0), but the indirect effect was significant (β_indirect effect_ = 0.343, 95%CI = [0.149, 0.564], not including 0). Specifically, the mediating effect of collective efficacy was significant (β = 0.210, 95%CI = [0.071, 0.378], not including 0). The mediating effect of risk perception was also significant (β = 0.133, 95%CI = [0.056, 0.235], not including 0), but smaller than collective efficacy (see Figure 4). Thus, collective efficacy and risk perception play a mediating effect between ethical attributes and consumers’ willingness to buy farmer-assisting agricultural products online. Accordingly, hypotheses H2b and H3b were verified.

The results of Study 1 and Study 2 showed that, compared with functional ethical attributes, symbolic ethical attributes have a more positive impact on consumers’ willingness to buy farmer-assisting agricultural products online, regardless of whether the image of farmers exists or not. Furthermore, ethical effects work through collective efficacy and risk perception, with collective efficacy playing a larger mediating role than risk perception. The first two studies briefly described the scenarios of farmer-assisting events, to explore the influence of ethical attributes on consumers’ willingness to buy farmer-assisting agricultural products online. However, these farmer-assisting event scenarios do not involve the expression and distinction of urgency. Thus, in Study 3, we studied how consumers’ willingness to buy farmer-assisting agricultural products online changes with the urgency of farmer-assisting events to explore the boundary conditions for the influence of ethical attributes on consumers’ willingness to buy farmer-assisting agricultural products online.

### Study 3: Moderating role of the urgency of farmer-assisting events

#### Pre-experiment

The purpose of the pre-experiment is to test the validity of different farmer-assisting event urgency scenarios and experimental materials. Referring to the study of Zheng et al. (2019), the farmer-assisting event urgency was divided into the sudden disaster scenario group and the normative difficulty scenario group. In this study, a random sample of 60 subjects (%females = 51.7%, M_Age_ = 30.237 years, SD = 1.213), was surveyed through the sample recommendation service of the Credamo platform. First, the subjects were invited to watch a push message: the first group read “An earthquake occurred in A area, and oranges are stagnant”; the second group read “The remote mountainous region in A area, and oranges are stagnant.” Then the semantic difference scale was used to rate the information on the urgency of the farmer-assisting event: “What do you think is the scenario in A area?” (1 = “sudden disaster scenario,” 7 = “normative difficulty scenario”). The results showed that there was a significant difference between the subjects’ ratings of the two groups [M_sudden_ = 3.438, SD = 1.848 vs. M_normative_ = 5.724, SD = 1.461; *t*(59) = −5.322, *p* < 0.001], and the stimulus for the urgency of the farmer-assisting event (i.e., the moderating variable) was tentatively considered valid. Experimental materials were selected from the same orange picture information in the Study 2 pre-experiment. The results showed that the subjects differed significantly [M_symbolic_ = 5.600, SD = 0.932 vs. M_functional_ = 2.133, SD = 1.074; *t*(58) = 13.350, *p* < 0.001] on different dimensions of ethical attributes, and Thus, the ethical attributes (i.e., independent variables) stimulus was valid. Finally, after the subjects read the above materials, they were asked to fill in the Situational Authenticity Scale (7-point Likert scale, 1 = “strongly disagree” and 7 = “strongly agree”), i.e., the degree to which this scenario conforms to the real scenario. The results of the One Sample t-test showed that the subjects rated the scenario authenticity significantly higher than 5 [M_scenario authenticity_ = 5.483, SD = 0.592, *t*(59) = 6.278, *p* < 0.001].

#### Formal experiments

The purpose of Study 3 is to test the moderating effect of the urgency of farmer-assisting events based on the previous two studies, that is, the moderating effect of the urgency of farmer-assisting events between ethical attributes, collective efficacy, and consumers’ willingness to buy farmer-assisting agricultural products online, as well as the moderating effect between ethical attributes, risk perception, and consumers’ willingness to buy farmer-assisting agricultural products online. A 2 (ethical attribute: functional ethical attribute vs. symbolic ethical attribute) × 2 (urgency of farmer-assisting events: sudden disaster scenario vs. normative difficulty scenario) between-group experimental design was designed. The formal experiment was carried out on the Cremado platform. A total of 180 subjects (%females = 52.2%, M_age_ = 30.240 years, SD = 6.89) were recruited, and the subjects were randomly assigned to one of 4 experimental scenario groups, namely sudden disaster × symbolic ethical attribute group, sudden disaster × functional ethical attribute group, normative difficulty × symbolic ethical attribute group, normative difficulty × functional ethical attribute group. First of all, the subjects read the urgency materials of farmer-assisting events in the pre-experiment, and then read the ethical attribute materials. After the four groups of subjects read the corresponding picture information, the subjects were asked to fill in the same questions as in Study 2, i.e., consumers’ willingness to buy farmer-assisting agricultural products online, collective efficacy, and risk perception. All items used the 7-point Likert scale for evaluation with 1 = “strongly disagree” and 7 = “strongly agree.” Finally, the demographic information was filled in. After excluding invalid questionnaires, there were 40 copies of each group, totaling 160 copies.

#### Analysis of results

First, a reliability test was performed. The results showed that the Cronbach’s alpha value of consumers’ willingness to buy farmer-assisting agricultural products online was 0.866, the Cronbach’s alpha value of collective efficacy was 0.882, and the Cronbach’s alpha value of risk perception was 0.815, all of which were greater than 0.7. Thus, each scale passed the reliability test.

Second, the results of the independent sample *t*-test showed that (see Table 6) the influence of symbolic ethical attributes and functional ethical attributes on consumers’ willingness to buy farmer-assisting agricultural products online [M_symbolic_ = 5.915, SD = 0.574 vs. M_functional_ = 5.598, SD = 0.449, *t*(158) = 3.865, *p* < 0.001], collective efficacy [M_symbolic_ = 5.806, SD = 0.950 vs. M_functional_ = 4.959, SD = 0.628, *t*(158) = 7.726, *p* < 0.001], and risk perception [M_symbolic_ = 3.338, SD = 0.712 vs. M_functional_ = 3.956, SD = 0.398, *t*(158) = −7.878, *p* < 0.001] were significantly different, that is, symbolic ethical attributes can stimulate consumers’ willingness to buy farmer-assisting agricultural products online, collective efficacy, and reduce risk perception more than functional ethical attributes. Thus, hypotheses H1, H2a, and H3a were verified again.

**Table 6:** Results of independent samples *t*-test.

Variable	Factors	Mean value	SD	T	95%CI	
					LLCI	ULCI
Consumers’ willingness to buy farmer-assisting agricultural products online	Symbolic ethical attribute	5.915	0.574	3.865^***^	0.155	0.478
	Functional ethical attribute	5.598	0.449			
Collective efficacy	Symbolic ethical attribute	5.806	0.429	7.726^***^	0.630	1.063
	Functional ethical attribute	4.959	0.628			
Risk perception	Symbolic ethical attribute	3.338	0.712	−7.878^***^	−0.774	−0.463
	Functional ethical attribute	3.956	0.398			

*** *p* < 0.001.

**Figure 5:**
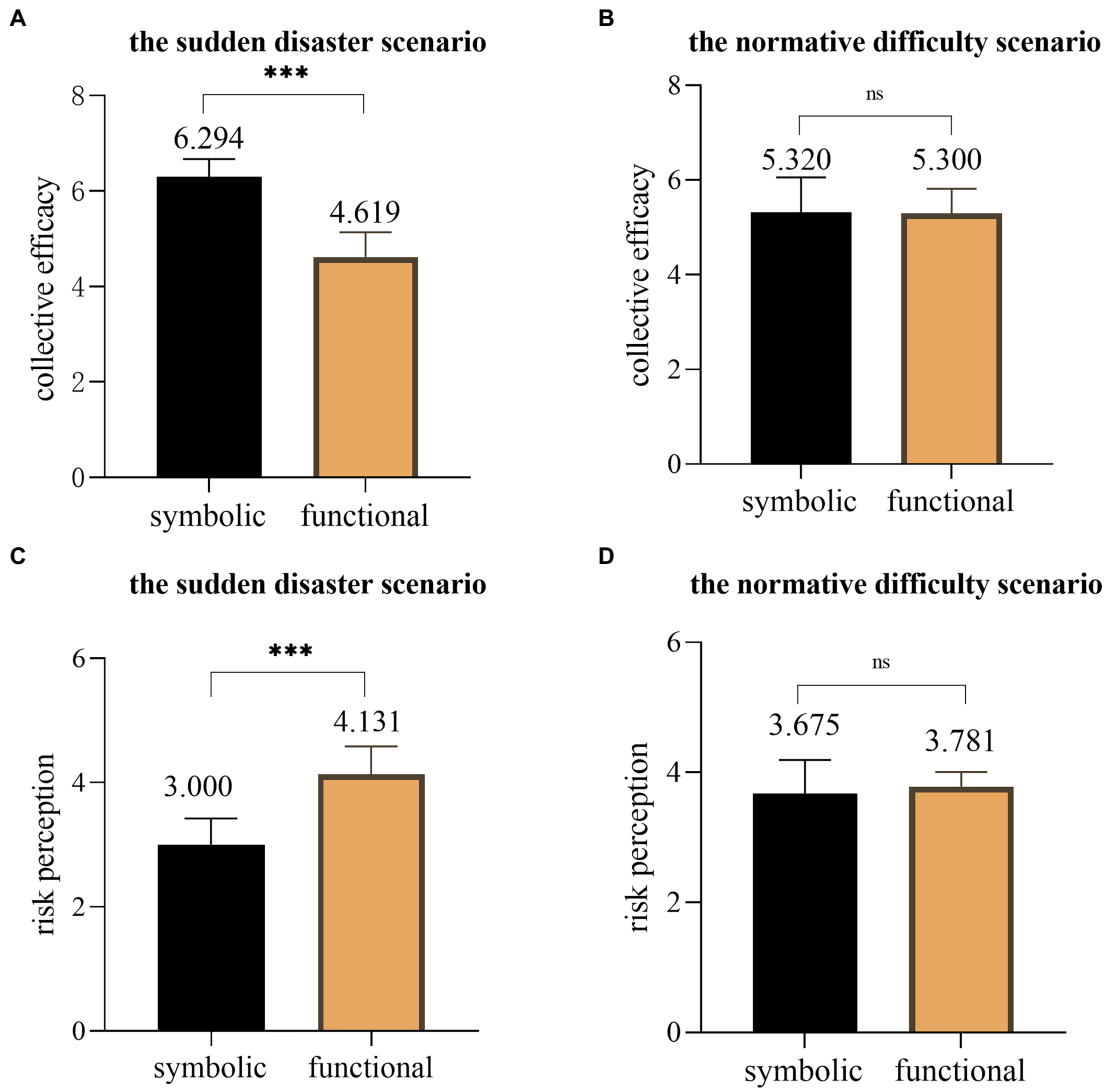
Multiple comparisons of symbolic ethical attributes with functional ethical attributes. **(A)** in the sudden disaster scenario, results of Study 3 on collective efficacy; **(B)** in the normative difficulty scenario, results of Study 3 on collective efficacy; **(C)** in the sudden disaster scenario, results of Study 3 on risk perception; **(D)** in the normative difficulty scenario, results of Study 3 on risk perception. ***p < 0.001; ns is not significant.

Then, the moderating effects of the farmer-assisting event urgency on the ethical attributes, collective efficacy, and consumers’ willingness to buy farmer-assisting agricultural products online, as well as moderating effects on ethical attributes, risk perception, and consumers’ willingness to buy farmer-assisting agricultural products online were examined. The results of the two-way analysis of variance (Two-way ANOVA) showed that the interaction item of urgency and morality of farmer-assisting events had a significant impact on collective efficacy, *F*(3, 156) = 64.204, *p* < 0.001. Further simple effect analysis (see Figure 5) found that in the sudden disaster scenario (group A), the collective efficacy of the subjects in the symbolic ethical attribute group (M_symbolic_ = 6.294, SD = 0.374) was significantly higher than that in the functional ethical attribute group (M_functional_ = 4.619, SD = 0.512, *p* < 0.001). In the normative difficulty scenario (group B), there was no significant difference in the collective efficacy between the symbolic ethical attribute group (M_symbolic_ = 5.320, SD = 0.726) and the functional ethical attribute group (M_functional_ = 5.300, SD = 0.447, *p* = 0.509). Similarly, the interaction term between the farmer-assisting event urgency and ethical attributes had a significant impact on risk perception, *F*(3, 156) = 50.966, *p* < 0.001. The results of the simple effect analysis (see Figure 3) showed that in the sudden disaster scenario (group C), the risk perception of the subjects in the symbolic ethical attribute group (M_symbolic_ = 3.000, SD = 0.418) was significantly lower than that in the functional ethical attribute group (M_functional_ = 4.131, SD = 0.447, *p* < 0.001). However, in the normative difficulty scenario (group D), there was no significant difference in risk perception between the symbolic ethical attribute group (M_symbolic_ = 3.675, SD = 0.510) and the functional ethical attribute group (M_functional_ = 3.781, SD = 0.225, *p* = 0.259).

In order to test the moderated mediation effect, we referred to the method proposed by Edwards and Lambert (2007) to test whether there is a difference in the size and significance of the mediation effect at the level of the moderation effect, to determine whether the mediation strength is moderated, so as to verify H4. Using process 3.3 of the statistical software SPSS22.0, the number of Bootstrap sampling was set to 5,000, the confidence interval was 95%, and model 7 (Hayes, 2013) was selected. The results showed that (see Table 7), the direct effect of ethical attributes and consumers’ willingness to buy farmer-assisting agricultural products online did not reach a significant level (β_direct effect_ = 0.080, 95%CI = [−0.179, 0.195], including 0) after adding collective efficacy and risk perception. In the normative difficulty scenario, the mediating effect of collective efficacy (β_indirect effect_ = 0.004, 95%CI = [−0.078, 0.058], including 0) and risk perception (β_indirect effect_ = 0.022, 95%CI = [−0.015, 0.065], including 0) did not reach a significant level. In the sudden disaster scenario, the mediating effect of ethical attributes → collective efficacy → consumers’ willingness to buy farmer-assisting agricultural products online (β_indirect effect_ = 0.354, 95%CI = [0.019, 0.684], not including 0), ethical attributes → risk perception → consumers’ willingness to buy farmer-assisting agricultural products online (β_indirect effect_ = 0.237, 95%CI = [0.061, 0.409], not including 0) reached a significant level, that is, H4 and H5 were verified. Thus, Study 3 further verified the boundary conditions for the existence of ethical effects by manipulating the urgency of farmer-assisting events.

**Table 7:** Test results of the moderated mediating effects based on Bootstrap.

Moderated variable	Mediated variable	Moderated level	Indirect effect	SE	95% CI	
					LLCI	ULCI
The urgency of farmer-assisting events	Collective efficacy	Sudden disaster scenario	0.354	0.178	0.020	0.709
		Normative difficulty scenario	0.040	0.033	−0.078	0.063
	Risk perception	Sudden disaster scenario	0.237	0.089	0.060	0.407
		Normative difficulty scenario	0.022	0.020	−0.015	0.065

## Conclusion and theoretical contributions

### Conclusion

This study begins with the socialization perspective of assisting farmers and aims to encourage the sustainable development of farmer-assisting agricultural consumption. Focusing on the ethical attributes of farmer-assisting agricultural products, we examine the effects of ethical attributes, the urgency of farmer-assisting events, and collective efficacy and risk perceptions on consumers’ willingness to buy farmer-assisting agricultural products online. This paper explains why many high-quality agricultural products in China are not widely welcomed and provides a theoretical foundation for e-commerce platforms and farmers to overcome market barriers.

Drawing on three experimental studies, this study found that consumers’ willingness to buy farmer-assisting agricultural products online was influenced by ethical attributes, while collective efficacy and risk perception mediate this effect, and that the urgency of farmer-assisting events moderates this effect.

### Theoretical contributions

First, by exploring the influence of the ethical attributes of farmer-assisting agricultural products on consumers’ willingness to buy farmer-assisting agricultural products online, this study expands the theoretical framework of the research on product ethical attributes and extends the application scope of ethical attributes in e-commerce-assisted agricultural marketing scenarios. Previous studies have proved that channelization strategy can effectively promote the sales of farmer-assisting agricultural products (Quan, 2021), but neglected the important role of product attributes in e-commerce-assisted agricultural marketing. The information on the ethical attributes of farmer-assisting agricultural products shows that they have the characteristics of farmer-assisting and poverty alleviation, which is different from the alternatives in other markets, and it has also become a key driver of consumers’ willingness to buy online. Thus, this study analyzes which ethical attribute (symbolic ethical attributes and functional ethical attributes) of products has a greater impact on online purchasing intentions when consumers buy farmer-assisting agricultural products online, to further clarify the effective ways for e-commerce enterprises to express ethics in farmer-assisting marketing. Specifically, compared with functional ethical attributes, symbolic ethical attributes have a more positive impact on consumers’ willingness to buy farmer-assisting agricultural products online. This conclusion is consistent with the conclusion of Zeng Q. Y. et al. (2021). At the same time, this conclusion is also in line with the existing practical problem in current e-commerce-assisted agricultural marketing, that is, emphasizing farmer-assisting but neglecting the quality characteristics.

Then, this study expands the antecedent psychological factors of consumers’ willingness to buy farmer-assisting agricultural products online from the perspective of collective efficacy and risk perception. Scholars have explained the reasons for consumers’ ethical consumption based on attribution theory, SOR theory, self-signaling theory, and other theories. They mainly focused on individual psychological variables such as the improvement of ethical sense at the level of ethical psychology (Zheng et al., 2019) and the trade-off of related interests at the level of teleological evaluation (Das et al., 2021). On the one hand, this study expands the psychological mechanism of consumers’ ethical consumption from the collective level. In the Internet context, new media communication can help consumers build and strengthen collective efficacy. Consumers may think that e-commerce platform companies need collective efforts to contribute to social good through the goal of farmer-assisting, and then translate this collective perception into positive purchasing intentions. On the other hand, different from the previous ethical consumption research that focused on offline physical consumption scenarios (Jin et al., 2020; Tofighi et al., 2020; Salam et al., 2022), this study is based on the e-commerce-assisted agricultural marketing scenario and introduces risk perception to analyze the key factors that inhibit consumers’ willingness to buy farmer-assisting agricultural products online. Based on this, the research on the potential mechanism affecting consumers’ ethical consumption willingness is further improved, and a more in-depth theoretical scenario exploration can be carried out on the psychological cognitive process of ethical consumption. The results of this study find that symbolic ethical attributes elicit higher levels of collective efficacy and lower risk perceptions in consumers than functional ethical attributes. Consumers’ collective efficacy and risk perception have dual mediating effects between ethical attributes and consumers’ willingness to buy farmer-assisting agricultural products online.

Finally, this study clarifies the boundary conditions for the mediating mechanisms of collective efficacy and risk perception in the process of ethical attributes influencing consumers’ willingness to buy farmer-assisting agricultural products online. Previous studies mainly emphasized that when the functional and ethical attributes of a product are weighed and compared simultaneously, consumers focus more on the functional attributes of the product (Luchs et al., 2012). In this study, we further find that for farmer-assisting agricultural products, symbolic ethical attributes can increase consumers’ willingness to buy farmer-assisting agricultural products online by increasing collective efficacy and reducing risk perceptions more than functional ethical attributes in the sudden disaster scenario, while there is no significant difference in normative difficulties. This study completes the research on the ethical attributes of products by introducing the variable of farmer-assisting event urgency.

## Management implications

After years of construction and development, e-commerce of agricultural products in China has become an engine and new impetus to boost the development of agricultural and rural economy. In the post-epidemic era, e-commerce of agricultural products in China is an essential and growing market segment. As we all know, New Oriental’s “Oriental Selection” platform has achieved remarkable achievements in marketing model innovation for farmer assistance. Thus, although the existing pain point of low standardization of fresh food remains unaddressed, operators should consider ethical positioning to boost consumers’ propensity to buy agricultural products online.

This study provides some theoretical guidance value and practical suggestions for e-commerce enterprises or marketers to effectively use the ethical attributes of agricultural products to enhance consumers’ willingness to purchase. First, when e-commerce companies carry out farmer-assisting marketing, they should pay attention to the information on the ethical attributes of farmer-assisting agricultural products. Specifically, the marketing strategy of enterprises should be based on the symbolic attributes of farmer-assisting agricultural products for public welfare and poverty alleviation, while taking into account the functional ethical attributes such as product quality and characteristics, to gradually form the market competitiveness and consumer stickiness of e-commerce farmer-assisting agricultural products. Secondly, e-commerce companies can consider the role of collective efficacy in consumers’ purchase decision-making process, and use Internet social media platforms to promote the dissemination of farmer-assisting events, to mobilize more netizens to participate in farmer-assisting instead of being a bystander. At the same time, enterprises should pay attention to the disclosure of information on real farmer-assisting events, improve the quality of farmer-assisting agricultural products, enhance the transportation and logistics system, and avoid the negative impact of reducing the willingness to buy farmer-assisting agricultural products due to consumers’ risk perception. Finally, e-commerce companies should pay attention to sudden disasters and help farmers in a timely manner when choosing what kind of situational marketing to help farmers. This is because consumers are more positively responsive to sudden disasters. They are more likely to provide timely relief activities for sudden disaster areas through farmer-assisting marketing activities.

## Research limitations and perspectives

This study uses a single stimulus (oranges) and farmer-assisting event urgency scenarios (sudden earthquakes and remote mountainous areas). farmer-assisting agricultural products include not only fruits but also vegetables, seafood, and other categories. There are also other types of emergency scenarios for farmer-assisting events, such as epidemics and high-temperature weather. Different product categories and events may lead to different conclusions. Future research could ultimately address these issues by replicating or testing extensions across multiple agricultural product categories.

Although this study focuses on the influence of ethical attributes on consumers’ willingness to buy farmer-assisting agricultural products online, it only lays emphasis on the process of consumers’ purchasing decisions. Due to uncertain factors such as product quality in the online shopping process, consumers’ expectations after purchasing may vary. It will affect the subsequent evaluation, repurchase, and other behaviors of consumption. Thus, the impact of consumer expectations on the evaluation of farmer-assisting agricultural products and the decision to repurchase can be considered in the future.

## Data availability statement

The original contributions presented in the study are included in the article/supplementary material, further inquiries can be directed to the corresponding author.

## Ethics statement

Written informed consent was obtained from the individual(s) for the publication of any potentially identifiable images or data included in this article.

## Author contributions

JW: conceptualization, methodology, formal analysis, and writing—original draft preparation. CW: software and validation. YY and CW: investigation. JW and CW: data curation. QZ: writing—review and editing and funding acquisition. All authors contributed to the article and approved the submitted version.

## Funding

This research was funded by Introduction of Talents of Minjiang University Science and Technology Pre-research Project “Research on the impact of Green Advertising Appeals on consumers’ Willingness to Pay Premium for Green Agricultural Products” and Major Project of Social Science Fund of Fujian Province in 2022 “Research on The Strategy of Fujian Province Adhering to Expanding Domestic Demand (FJ2022Z006)”.

## Conflict of interest

The authors declare that the research was conducted in the absence of any commercial or financial relationships that could be construed as a potential conflict of interest.

## Publisher’s note

All claims expressed in this article are solely those of the authors and do not necessarily represent those of their affiliated organizations, or those of the publisher, the editors and the reviewers. Any product that may be evaluated in this article, or claim that may be made by its manufacturer, is not guaranteed or endorsed by the publisher.
